# Wide-Dynamic-Range Lead-Free SWIR Image Sensors Based on InAs Thin-Film Quantum-Dot Photodiodes †

**DOI:** 10.3390/s25237345

**Published:** 2025-12-02

**Authors:** Myonglae Chu, Wenya Song, Joo Hyoung Kim, Tristan Weydts, Vladimir Pejovic, Jiwon Lee, Minhyun Jin, Sang Yeon Lee, Yoora Seo, Hyunyoung Yoo, Jonas Bentell, Abu Bakar Siddik, Isabel Pintor Monroy, Marina Vildanova, Arman Uz Zaman, Tae Jin Yoo, Antonia Malainou, Wagdy Hussein, Annachiara Spagnolo, Gauri Karve, Itai Lieberman, Stefano Guerrieri, Pawel E. Malinowski

**Affiliations:** 1 Interuniversity Microelectronics Centre (IMEC), Kapeldreef 75, 3001 Leuven, Belgium; wenya.song@imec.be (W.S.); joohyoung.kim@imec.be (J.H.K.); tristan.weydts@imec.be (T.W.); vladimir.pejovic@imec.be (V.P.); jiwon.lee@imec.be (J.L.); minhyun.jin@imec.be (M.J.); sangyeon.lee@imec.be (S.Y.L.); yoora.seo.ext@imec.be (Y.S.); hyunyoung.yoo.ext@imec.be (H.Y.); abu.bakar.siddik@imec.be (A.B.S.); isabel.pintormonroy@imec.be (I.P.M.); marina.vildanova@imec.be (M.V.); arman.uzzaman@imec.be (A.U.Z.); tae.jin.yoo@imec.be (T.J.Y.); antonia.malainou@imec.be (A.M.); wagdy.hussein@imec.be (W.H.); annachiara.spagnolo@imec.be (A.S.); gauri.karve@imec.be (G.K.); itai.lieberman@imec.be (I.L.); stefano.guerrieri@imec.be (S.G.); pawel.malinowski@imec.be (P.E.M.); 2 Department of Semiconductor Engineering, Pohang University of Science and Technology (POSTECH), Pohang 37673, Republic of Korea; 3 Department of Photonics and Nanoelectronics, Hanyang University, Seoul 04763, Republic of Korea; 4 Department of Electronic Engineering, Seoul National University of Science and Technology, 232 Gongneung-ro, Seoul 01811, Republic of Korea

**Keywords:** CMOS image sensor, InAs quantum dot photodiode, short-wavelength infrared (SWIR), thin-film photodiode (TFPD)

## Abstract

This work presents a monolithically integrated short-wavelength infrared (SWIR) image sensor based on indium arsenide (InAs) quantum dot photodiodes (QDPDs). The thin-film photodiode (TFPD) architecture enables direct integration on silicon readout integrated circuits (ROICs), eliminating wafer-to-wafer bonding and providing a scalable, RoHS-compliant alternative to lead-based colloidal quantum dot (CQD) devices. The proposed 3T pixel design incorporates dual conversion gain (DCG), enabling wide dynamic range imaging. The fabricated prototype achieves external quantum efficiencies of 28% at 1200 nm and 4.8% at 1400 nm, together with a dynamic range of 83.5 dB. A frame-based digital correlated double sampling (CDS) scheme stores the reset level in the digital domain and subtracts it after integration, thereby suppressing reset kTC noise and mitigating random telegraph signal (RTS) noise. Imaging demonstrations highlight SWIR-specific functionalities, including material discrimination, imaging through smoke, and transmission through silicon wafers. A performance comparison with previously reported SWIR pixels further confirms the competitiveness of the proposed InAs QDPD imager. These results establish InAs QDPDs as a promising platform for next-generation SWIR imaging, combining high sensitivity, extended spectral coverage, and scalable integration.

## 1. Introduction

**Figure 1:**
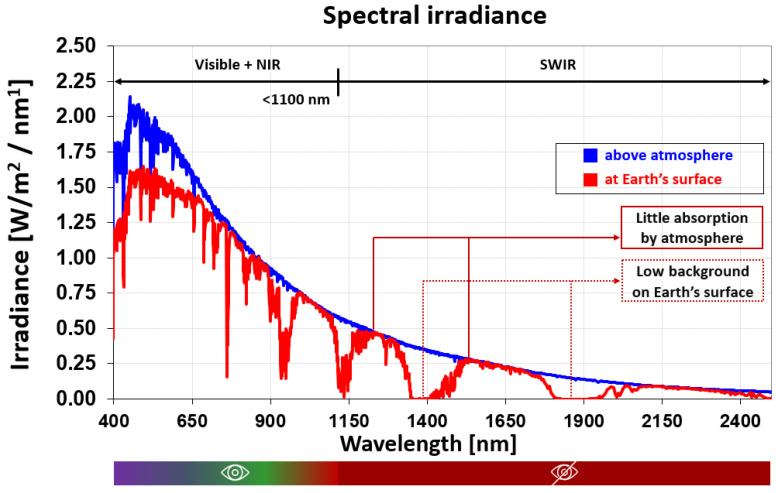
Solar spectral irradiance above the atmosphere (Blue) and at the Earth’s surface (Red); The bar below the x-axis indicates the observable spectral range: the eye icon represents the visible (VIS) and near-infrared (NIR) ranges, and the stylized eye icon represents the short-wave infrared (SWIR) range, where special detection is often required. Data: ASTM G173-03 (via NREL AM1.5 Reference Spectra) [2].

The demand for image sensors in the short-wavelength infrared (SWIR) range (commonly defined as 1100–2000 nm) has been steadily increasing due to their superior sensitivity compared with silicon photodiodes in applications such as medical imaging, security, automotive vision, and industrial inspection. This growth is mainly driven by the unique atmospheric transmission characteristics of the SWIR spectral range [1], as illustrated in Figure 1 [2]. Certain wavelengths, particularly around 1200 nm, 1550 nm, and 2000 nm, correspond to high-transmission atmospheric windows where absorption by water vapor and other gases is minimal, enabling high signal-to-noise ratio (SNR) imaging. In biomedical applications, these wavelengths allow deeper photon penetration into biological tissue, enhancing contrast and spatial resolution for non-invasive spectroscopy, functional imaging, and image-guided surgery [3,4]. Conversely, strongly absorbed bands near 1400 nm and 1900 nm experience minimal solar background at the Earth’s surface due to atmospheric absorption. When using active illumination at these wavelengths, the negligible ambient light enables exceptionally high-SNR detection, making them valuable for active sensing tasks such as material discrimination and object detection in adverse weather conditions [5,6,7].

Conventional SWIR image sensors typically rely on Die-to-Wafer (D2W) bonding to connect III–V photodiode arrays to silicon readout integrated circuits (ROICs) [5]. While this approach achieves high performance, it presents significant challenges for scaling and cost reduction due to the complexity of the bonding process. Alignment and bonding can introduce defects, reduce yield, and increase fabrication costs, limiting large-scale production. To address these challenges, we propose a SWIR sensor architecture that employs monolithic fabrication of thin-film photodiodes (TFPDs) directly on the silicon readout wafer [6,7]. This approach eliminates the bonding process and simplifies the fabrication process. Monolithic integration reduces the number of processing steps, minimizes misalignment risks, and improves yield, thereby enabling scalable manufacturing and supporting the development of new form factors.

**Figure 2:**
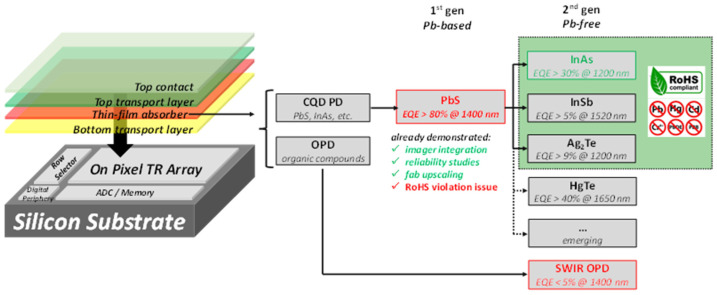
SWIR imager options highlighting 2 generations of QD sensor: PbS-based (1st gen) and Pb-free (2nd gen) [7].

In the early development of TFPD-based colloidal quantum dot (CQD) SWIR image sensors, lead sulfide (PbS) is the most widely used material due to its high external quantum efficiency (EQE, >80%). However, PbS contains lead (Pb), a toxic element regulated under the Restriction of Hazardous Substances (RoHS) directive, which limits its use in consumer, automotive, and medical electronics. This has motivated research into Pb-free alternatives, as summarized in Figure 2. Organic photodiodes (OPDs) have been explored as one option. Although their spectral response can extend to ~1400 nm, the EQE in this region is typically <5% at 1400 nm [8], making them unsuitable for long-wavelength SWIR applications.

**Figure 3:**
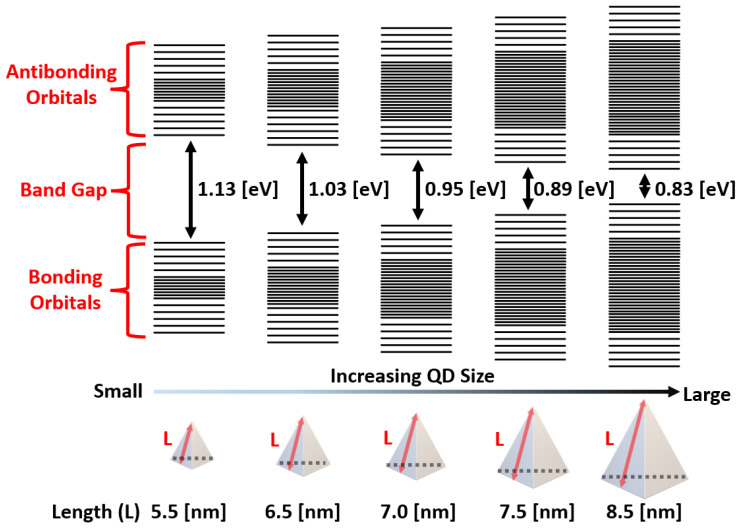
Dependence of energy bandgap on InAs quantum dot size.

In this work, we employ indium arsenide (InAs) and indium arsenide phosphide (In(As,P)) quantum dots (QDs) as RoHS-compliant CQD materials. The InAs QDPD developed in this study, with an average size of approximately 6.5 nm, is designed with a target wavelength around 1200 nm, while the In(As,P) QDPD, whose structure and average size of about 7.4 nm have been reported in [9], is tailored for a target wavelength near 1400 nm. Figure 3 illustrates the bandgap tunability of the InAs QD used in this work. These materials offer broad and precise bandgap tunability through control of particle size and composition, enabling sensors optimized for different SWIR wavelength ranges. This tunability, combined with environmental compliance, makes InAs-based QDs an attractive material platform for next-generation SWIR sensors. Based on the proposed InAs QDPD, the key considerations and potential trade-offs associated with employing InAs QDPDs were analyzed, including the effects of carrier type selection and interfacial charge transport. Furthermore, because no photodiode is formed in the silicon region in the TFPD architecture, this area can be used for additional circuitry. In this work, it is utilized to implement dual conversion gain (DCG) capacitors and extra switches within the pixel, enabling high dynamic range (DR) operation. The DCG architecture improves performance under both low-light and high-light conditions.

In summary, we have developed a monolithically integrated DCG InAs QD SWIR pixel that achieves external quantum efficiencies of approximately 30% at 1200 nm and 5% at 1400 nm (In(As,P) QD), with a measured dynamic range of 83.5 dB.

## 2. Quantum Dot Photodiode (PD) and Image Sensor Design

### 2.1. Imager Fabrication with InAs Quantum Dot PD

**Figure 4:**
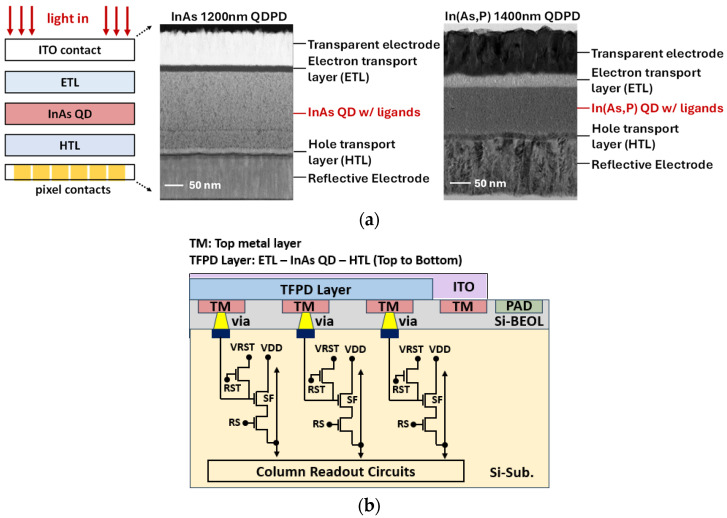
(**a**) InAs and In(As,P) QD Photodiode stack structure; (**b**) The simplified pixel architecture of QDPD on silicon readout circuit.

Figure 4a shows the schematic of the QD device structure used in the fabricated QDPD. The device employs a top-illuminated architecture in which incident light enters through a transparent indium tin oxide (ITO) contact layer, enabling efficient optical transmission into the underlying layers. Directly beneath the ITO lies a carrier transport stack composed of electron (ETL) or hole (HTL) transport layers, which selectively guide photo-generated carriers toward their respective electrodes. In this configuration, signal charges are collected and stored in the pixel circuit, while carriers of the opposite polarity are efficiently drained toward the ITO side. The central active layer consists of QDs capped with organic ligands, forming a uniform and densely packed film. The bandgap of the QDs is engineered to provide strong absorption in the SWIR range, targeting 1200 nm and 1400 nm in this work. The total stack thickness is optimized to balance optical absorption, carrier transport efficiency, and capacitance, supporting high EQE and low-noise operation. For the 1200 nm QDPD stack, a 15 nm HTL was deposited on the electrode using the physical vapor deposition (PVD) method, followed by spin-coating a 200 nm InAs QD layer. Subsequently, a 20 nm ETL and a 100 nm ITO layer were deposited by PVD. For the 1400 nm, a QDPD stack was fabricated following the same process sequence, except that a 100 nm In(As,P) QD layer and a 40 nm ETL were used.

### 2.2. Pixel Circuit and Imager Design: Electron-(e2ROIC) and Hole-Collection (h2ROIC)

Figure 4b presents a simplified schematic of the sensor architecture implemented in a 3T pixel structure. The TFPD is connected through the top metal (TM) to the source follower in each pixel, thereby forming the pixel array.

**Figure 5:**
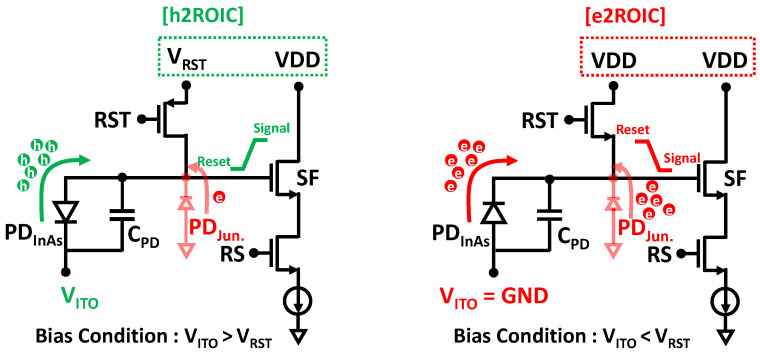
Pixel circuit configurations for hole-collection (h2ROIC, **left**) and electron-collection (e2ROIC, **right**) modes.

The signal type of the QDPD is determined by the arrangement of the transport layers, enabling either electron- or hole-collection operation. For example, when the HTL is positioned between the ITO contact and the QD layer, and the ETL is located beneath the QD layer, the pixel operates in electron-collection mode. To read out the pixel signal, the bias configuration is adjusted accordingly. In electron-collection (e2ROIC), the ITO voltage (V_ITO_) is set lower than the reset voltage V_RST_, typically with V_ITO_ = 0 V (ground) and V_RST_ = VDD. Conversely, in hole-collection (h2ROIC), V_ITO_ is set higher than V_RST_. With these conditions, the desired photo-generated carriers are collected at the pixel, as illustrated in Figure 5.

The e2ROIC pixel circuit operates identically to a conventional 3T pixel in a standard silicon image sensor. Because the reset voltage can share a single power line with the pixel supply (VDD), this configuration is favorable for pixel scaling. However, the high reset voltage increases the junction current of the reset transistor, which is then added to the intrinsic dark current of the photodiode, resulting in a higher total dark current. In contrast, the h2ROIC configuration applies a lower reset voltage to the reset transistor junction diode, reducing this additional leakage current. The trade-off is that h2ROIC requires an extra metal line, since its reset voltage cannot share the pixel supply, and it also requires the reset transistor to be implemented as a PMOS device to provide a blooming path. Consequently, h2ROIC is less favorable for more aggressive pixel scaling as compared to e2ROIC.

**Figure 6:**
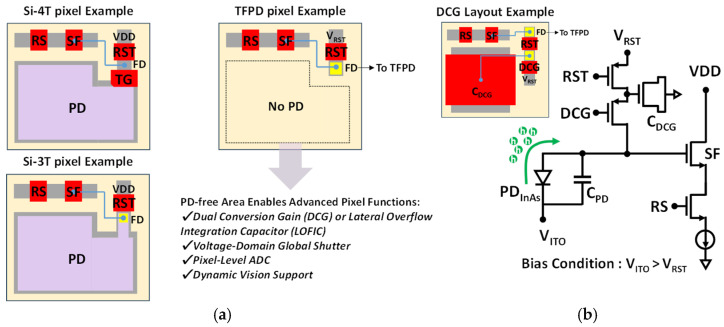
(**a**) Comparison of conventional CMOS image sensor pixel layouts (**left**) with integrated photodiode (PD) regions and the proposed thin-film photodiode (TFPD) pixel layout (**right**) without a silicon-based PD. RS: row selection transistor, SF: source follower, RST: reset transistor, TG: transfer gate, FD: floating diffusion. (**b**) Schematic and layout of the hole-collection InAs QDPD DCG pixel.

In a TFPD pixel design, no photodiode is formed in the silicon area, as shown in Figure 6a, which allows this region to be used for additional functionality. For example, by adding an extra capacitor and transistor, the TFPD can be adapted to support advanced features such as DCG, lateral overflow integration capacitor (LOFIC), or voltage-domain global shutter operation. Alternatively, multiple transistors can be added to enable pixel-level circuits such as ADCs or dynamic vision sensor (DVS) pixels. In this work, the silicon area was utilized to integrate a DCG capacitor and switch, enabling the realization of a hole-collection InAs QDPD 3T DCG pixel [10], as shown in Figure 6b.

## 3. Characterization Results

### 3.1. Sensor Characteristics

**Figure 7:**
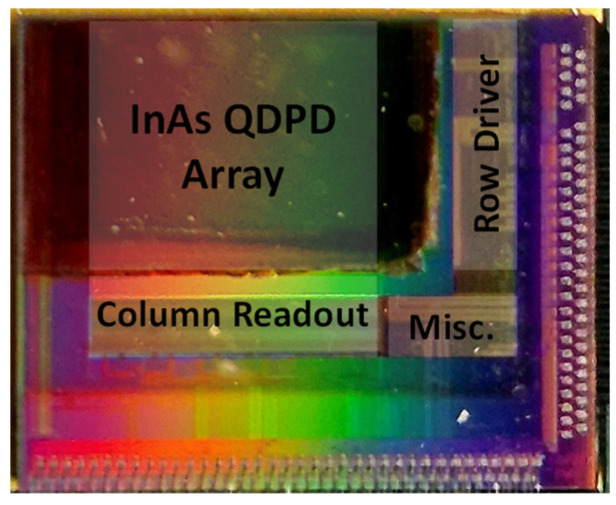
The micrograph of the prototype InAs QDPD Image Sensor.

The micrograph of the prototype chip is shown in Figure 7, illustrating the physical view and monolithic integration of the TFPD pixel array on the silicon substrate. For the 5 µm pitch pixel, the high-conversion-gain (HCG) mode is defined by the intrinsic capacitance of the InAs QDPD, which is approximately 20 fF. In the low-conversion-gain (LCG) mode, the total capacitance increases to 108 fF, corresponding to the sum of the HCG capacitance and the additional dual-conversion-gain capacitor (C_DCG_). The sensor operates in a standard sequential readout mode with a frame rate of approximately 19 frames per second (fps). In the hole-collection configuration, the pixel reset voltage (V_RST_) is set to 1.8 V to initialize the photodiode anode node (V_PD_), while the ITO contact at the photodiode cathode (V_ITO_) is biased at 5 V. This biasing scheme ensures that the InAs QDPD operates under optimal reverse bias conditions for hole collection, thereby maintaining high sensitivity and maximizing the achievable dynamic range.

**Figure 8:**
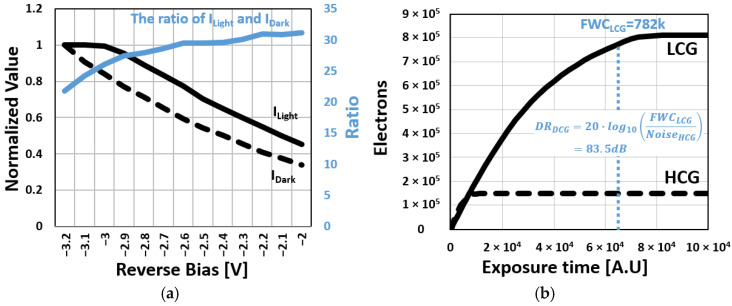
(**a**) Measured current under illumination (ILight), dark current (IDark), and the ratio, with bias voltage; (**b**) Measured photon response curve of InAs QDPD DCG pixel.

To evaluate the photoresponse characteristics of the photodiode, Figure 8a presents the normalized ratio of the measured current under illumination (I_Light_) and a dark current (I_Dark_) as a function of bias voltage, measured under a fixed illumination condition corresponding to an irradiance of approximately 2.3 mW/cm^2^ at the sensor surface. The illumination was provided by a 150 W halogen lamp, and only the wavelength of 1200 nm was selected and delivered to the sensor surface through a monochromator. Each current was normalized to its respective maximum value, while the I_Light_/I_Dark_ ratio was calculated using absolute current values. As photon-induced charge accumulated, the reverse bias across the InAs QDPD was reduced, leading to a gradual decrease in both I_Light_ and I_Dark_. Nevertheless, the ratio of I_Light_ and I_Dark_ remained sufficiently high to support a signal increase. This behavior is similar to that of logarithmic-response pixels [11], where reduced I_Light_ at higher signal levels effectively extends the dynamic range. The measured response curve in Figure 8b further confirms that the proposed pixel achieves a wide dynamic range of 83.5 dB. The full-well capacity (FWC), defined as the variance peak point in the photon transfer curve (PTC), is measured at 71.9 ke^−^ in HCG mode and 782 ke^−^ in LCG mode. This broad dynamic range allows the sensor to capture scenes under both low- and high-light conditions.

**Figure 9:**
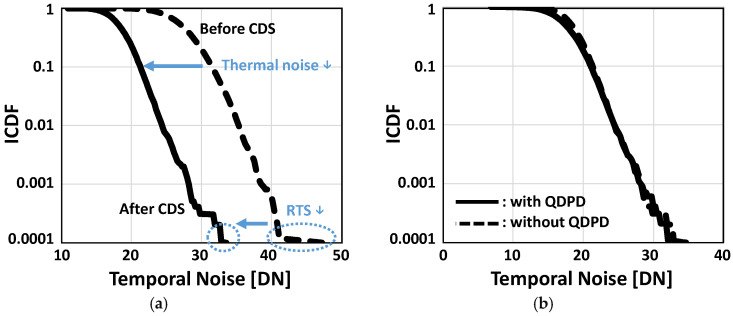
(**a**) Inverse cumulative distribution function (ICDF) before and after CDS; (**b**) RTS performances comparison with and without InAs QDPD.

The noise performance of the sensor is analyzed in Figure 9 using the inverse cumulative distribution function (ICDF). To suppress noise, the sensor employs a frame-based digital correlated double sampling (CDS) technique. This effectively reduces reset kTC noise, which is the dominant dark noise source in conventional 3T pixels, and also mitigates random telegraph signal (RTS) noise. As shown in Figure 9a, the thermal noise level is reduced from 32 to 21 digital numbers (DN), and the RTS noise tail in the ICDF plot is significantly diminished. Additional analysis comparing the sensor’s measured noise characteristics with and without the InAs QDPD (Figure 9b) was performed to verify the contribution of the QDPD itself to RTS noise. Although quantum dot photodiodes are generally expected to exhibit numerous trap states that can induce RTS noise, the proposed pixel shows no observable RTS noise originating from the InAs QDPD. Instead, the RTS noise is dominated by the source follower, resulting in negligible differences in the ICDF tails with and without the QDPD present.

**Figure 10:**
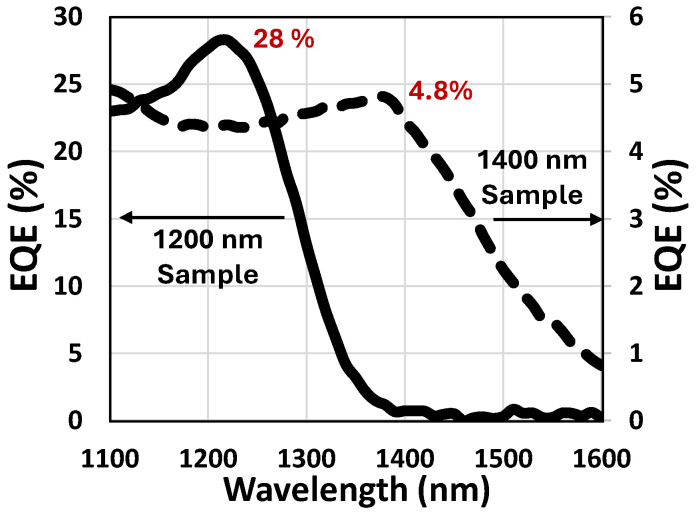
Measured EQE of two different stacks (for peak EQE @ 1200 (InAs) nm and 1400 (In(As,P)) nm) versus wavelength.

The measured external quantum efficiency (EQE) results are summarized in Figure 10. Two photodiodes were fabricated, each designed for different target wavelengths, 1200 nm and 1400 nm, corresponding to InAs and In(As,P) QD-based devices, respectively. The devices achieved EQEs of 28% at 1200 nm and 4.8% at 1400 nm. Figure 10 also demonstrates the inherent wavelength tunability of QDPDs, which can be engineered to cover a broad spectral range, from the visible to the short-wave infrared, by adjusting the quantum-dot size and composition. However, for long-wavelength detection, larger quantum dots are required, which reduces the bandgap energy. This reduction, in turn, increases dark current and decreases EQE due to enhanced thermal generation. Finally, Table 1 summarizes the key performance parameters of the proposed InAs QDPD pixel, confirming its competitiveness as a candidate for next-generation SWIR imaging applications.

### 3.2. SWIR Image Demonstration

**Figure 11:**
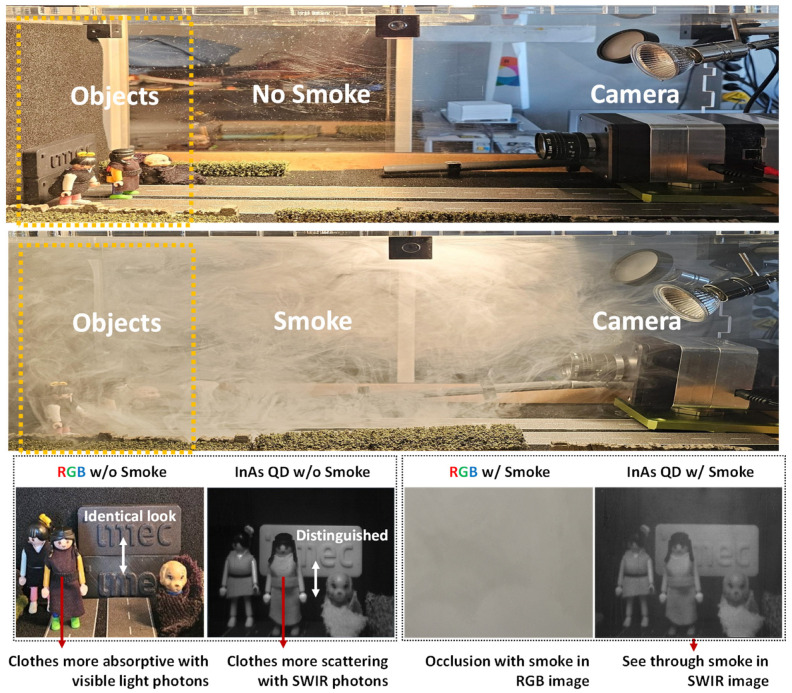
RGB and 1200 nm wavelength sample images captured w/ and w/o smoke conditions.

The captured sample images in Figure 11 demonstrate the clear advantages of the proposed InAs SWIR sensor over conventional RGB imaging under both clear and obscured viewing conditions, using illumination from a 500 W tungsten halogen lamp and an exposure time of approximately 150 µs. In the smoke-free case, both RGB and SWIR images depict the overall scene; however, the InAs SWIR sensor further reveals material-dependent differences that are not detectable in RGB. For instance, garments composed of different fabrics, which appear visually identical in RGB, exhibit distinct scattering characteristics under SWIR illumination, thereby enabling straightforward material discrimination.

In the smoke-filled case, the benefits of the InAs SWIR sensor become even more apparent. The RGB image is severely degraded by light scattering from the smoke, which makes objects nearly invisible. In contrast, the InAs SWIR sensor maintains high contrast and preserves fine detail, allowing objects to remain clearly visible through the smoke. This enhanced penetration capability arises from the longer wavelengths of SWIR light, which undergo significantly less scattering compared with visible light. Remarkably, the sensor maintains this performance despite a relatively high PRNU.

**Figure 12:**
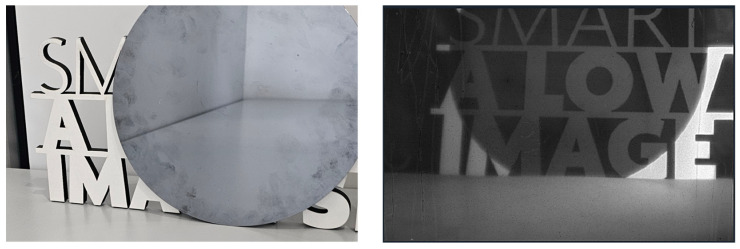
RGB and 1200 nm wavelength sample images captured w/ (**Left**) and w/o (**Right**) silicon wafer.

Further demonstrating the unique strengths of the InAs SWIR sensor, Figure 12 presents an experiment in which imaging was performed through a silicon wafer, using the same illumination source as in Figure 11. At visible wavelengths (RGB), silicon is completely opaque, preventing any visualization of objects behind the wafer. However, the InAs SWIR sensor clearly reveals the hidden objects, since silicon is highly transparent in this spectral region. This property highlights the value of SWIR imaging for applications such as through-silicon inspection, semiconductor wafer alignment, and packaging verification.

These test results demonstrate the strong potential of the proposed InAs SWIR sensor beyond the limits of conventional RGB imagers, enabling functionalities such as material discrimination, visibility through smoke and other obscurants, and imaging through silicon or other semi-transparent materials at SWIR wavelengths. For reference, Table 2 summarizes a performance comparison with recently reported SWIR pixels [5,12,13,14,15]. The comparison shows that the presented InAs QDPD achieves a competitive balance of pixel pitch, spectral coverage, EQE, and dynamic range, while maintaining RoHS-compliant material composition.

## 4. Conclusions

These proof-of-concept imagers represent a significant step forward in advancing Quantum Dot (QD) Short-Wave Infrared (SWIR) technology with non-restricted (RoHS-compliant) materials. The experimental results highlight the strong potential of InAs QDPDs to deliver high external quantum efficiency and a wide dynamic range. The integration of noise reduction techniques, including frame-based digital correlated double sampling and hole-collection readout architectures, has proven effective in suppressing reset kTC noise, random telegraph signal noise, and dark current, thereby enhancing overall image quality. Beyond device-level performance, the demonstrated SWIR imagers showcase distinctive advantages such as the ability to penetrate smoke and to distinguish between materials regardless of visible color, functionalities that conventional visible image sensors cannot provide. The monolithic InAs QDPD platform offers a scalable route toward high-resolution, wide-dynamic-range SWIR imagers. Its compatibility with CMOS readout and RoHS-compliant composition positions it as a strong candidate for next-generation applications in automotive perception, biomedical imaging, and advanced industrial inspection. These perspectives highlight the broader impact of this work, reinforcing the role of InAs QDPDs as a promising approach for future SWIR imaging technologies.

## Figures and Tables

**Table 1:** Measured image sensor performance. Except for DC, DSNU, and PRNU, all results are from 1200 nm samples.

Parameter	Value	Unit
Technology	0.13	[µm]
Pixel Size	5	[µm]
Pixel Resolution	768 × 512	[Mp]
Dark Current (DC) (1200/1400 nm)	23.6/80	[ µ A/cm^2^]
Dark Read Noise (HCG/LCG)	52.2/276	[e-]
Conversion Gain (HCG/LCG)	8.13/1.48	[ µ V/e-]
FWC (HCG/LCG)	71.9 k/782 k	[e-]
PRNU (1200/1400 nm)	8.1/3.5	[%]
DSNU (1200/1400 nm)	18.2/50.8	[e-]
Dynamic Range (HCG/LCG/DCG)	62.8/69/83.5	[dB]

All measurements were performed at room temperature.

**Table 2:** Pixel performance comparison table.

Parameter	This Work ^(1)^		[5]	[12]	[13]	[14]	[15] ^(2)^	
Photodiode	InAs QD		InGaAs	PbS QD	PbS QD	Ge-on-Si	InAs QD	
Pixel Pitch [µm]	5		5	7	1.62/2.2	10	-	
Spectral Range [nm]	400–1600		400–1700	400–1700	1400	850–1400	400–1600	
Peak wavelength [nm]	1200	1400 ^(3)^	-	-	-	-	940	1400
Dark Current [µA/cm^2^]	23.6	80	* 0.0025	<0.005	** 0.3/0.51	-	* 0.02	* 100
Peak EQE [%]	28	4.8	-	15–45	* 60	-	* 39	* 15
PRNU [%]	8.1	3.5	-	-	1.4/0.7	-	-	-

* Values read from the graph; ** Recalculated values from the data; ^(1)^ Results with −3.2 V bias voltage; ^(2)^ Results with −1.5 V bias voltage. ^(3)^ In(As,P) Photodiode.

## Data Availability

Data is contained within the article.
